# Species identification for Indian seafood markets: A machine learning approach with a fish dataset

**DOI:** 10.1016/j.dib.2024.111209

**Published:** 2024-12-09

**Authors:** Priyanka Paygude, Namita Shinde, Amol Dhumane, Geeta S. Navale, Prashant Chavan, Atul Kathole, Vijaykumar Bidve

**Affiliations:** a Bharati Vidyapeeth (Deemed to be University) College of Engineering, Pune, India; b Symbiosis Institute of Technology, Symbiosis International (Deemed University), Pune, India; c Sinhgad Institute of Technology and Science, Pune, India; d Dr. D. Y. Patil Institute of Technology, Pimpri, Pune, India; e School of CSIT, Symbiosis Skills and Professional University, Kiwale, Pune, India

**Keywords:** Fish classification, Fish dataset, Fish detection, Machine learning

## Abstract

The Indian sea fish market is a dynamic and significant sector, contributing to both the domestic economy and the global seafood trade. This fish dataset is specifically curated for machine learning applications in the Indian seafood industry. It includes a comprehensive collection of images representing eight commercially significant fish species native to Indian waters, comprising a total of 8488 images. This dataset serves as a valuable resource for developing and refining machine learning models in the domain. It focuses on the eight most widely consumed fish species in India: Pomfret (scientific name: Pampus argeneus, local name: Paplet), Mackerel (scientific name: Scomber scombrus, local name: Bangda), Black Snapper (scientific name: Apsilus dentatus, local name: Tilapia), Indian Carp (scientific name: Cyprinus carpio, local name: Rohu), Prawn (scientific name: Dendrobranchiata, local name: Kolambi), Pink Perch (scientific name: Zalembius rosaceus, local name: Rani Fish), Indian flathead (scientific name: Lates calcarifer, local name: Bhetki Fish) and Black Pomfret (scientific name: Parastromateus niger, local name: Black Paplet). Article details the data acquisition process, and metadata structure, ensuring accessibility and usability for researchers and industry professionals. This resource facilitates the development of machine learning models for tasks such as species identification within the Indian seafood industry.

Specifications Table

**Table:** 

Subject	Machine Learning, Animal Science, Food Science.
Specific subject area	Fish dataset with quality classification
Type of data	Images
Data collection	The eight most widely consumed seafood fish species in India included in this dataset are Pomfret (*Pampus argenteus*, locally known as Paplet), Mackerel (*Scomber scombrus*, locally known as Bangda), Black Snapper (*Apsilus dentatus*, locally known as Tilapia), Indian Carp (*Cyprinus carpio*, locally known as Rohu), Prawn (*Dendrobranchiata*, locally known as Kolambi), Pink Perch (*Zalembius rosaceus*, locally known as Rani Fish), Indian Flathead (*Lates calcarifer*, locally known as Bhetki Fish), and Black Pomfret (*Parastromateus niger*, locally known as Black Paplet). The images of each species are meticulously organized into separate folders for streamlined access and usability. We made a concerted effort to capture images of the fish from multiple angles, under varying lighting conditions, and against diverse backgrounds to ensure a comprehensive and diverse dataset. The images were saved as JPEG files, uniformly scaled to a consistent resolution.
Data source location	Fish Market, Ganesh Peth, Pune, Maharashtra, India Longitude and Latitude: N 18° 30.7018′ , E 73° 51.8634′
Data accessibility	Repository name: Indian Seafood Fish Dataset Data identification number: 10.17632/mxf2c45yb5.1 Direct URL to data: https://data.mendeley.com/datasets/mxf2c45yb5/1
Related research article	*None*

## 1 Value of the Data

• India is a significant contributor to global seafood exports, playing a crucial role in the industry's economic landscape. The market achieved significant volume of a 20.7 million tons in 2023 and is expected to nearly double by 2032, driven by factors such as rising incomes and growing awareness of the health benefits of seafood.
• Researchers can utilize this fish dataset to test and compare the performance of existing fish classification algorithms. This facilitates evaluates how effectively various algorithms perform on a standardized dataset.
• Researchers can use existing fish image datasets to train new machine learning models for tasks such as fish species identification, object detection, or abundance estimation. By assessing their model's performance on a known dataset, researchers can validate their approach and confirm its effectiveness.
• By training computer vision models to identify fresh fish from images, these models can be deployed in various scenarios to ensure consumers receive high-quality fish.

## 2 Background

The Indian sea fish market is a significant sector with immense potential for growth. With the evolution of consumer preferences and advancements in technology, this industry is well-positioned for sustained development, thereby contributing notably to the nation's food security and economic prosperity. With this motivation, this fish dataset is compiled to cover most commercially viable species within the Indian Seafood industry. This dataset of fish images can be utilized to train machine learning models to automatically identify fish species from photographic inputs, which may prove beneficial for marine research and underwater exploration. Furthermore, the fish dataset is applicable to a range of broader computer vision tasks, including object detection and image segmentation, thereby enhancing the automation of fish detection and classification processes. Fish researchers and the fishing industry can benefit by saving a significant amount of time and effort using AI models compared to manual methods. This technological advancement enables customers to find products visually, such as image based search on web.

## 3 Data Description

The dataset includes images of the eight most widely consumed local species of fresh fish, ensuring the models trained on it can effectively address the complexities encountered in real-world applications. These images feature a wide variety of backgrounds, lighting conditions, camera angles and high-resolution images (1024 × 768 pixels at 72 dpi) capturing intricate details of the fish.

**Fig. 1:**
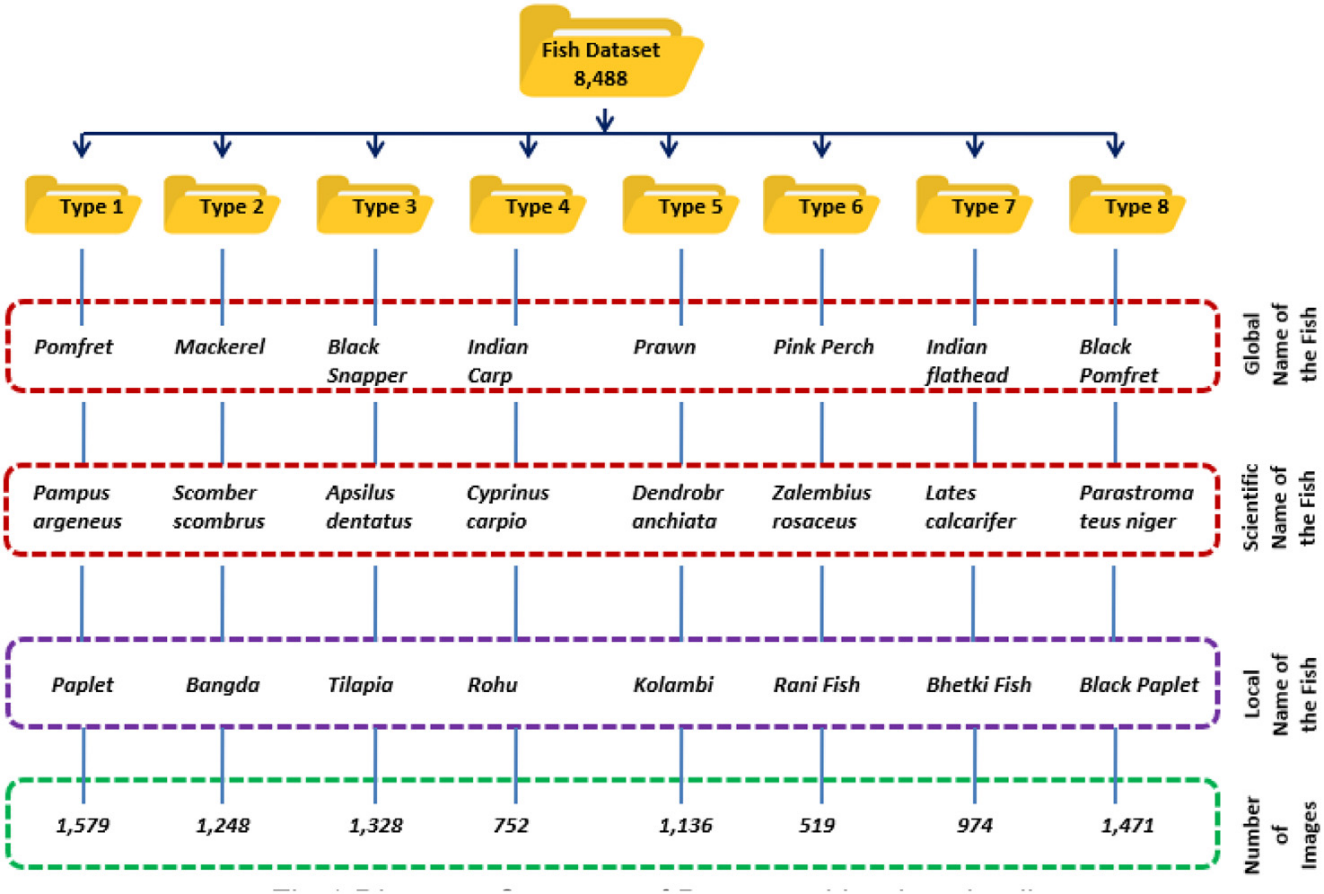
Directory structure of dataset with other details.

**Fig. 2:**
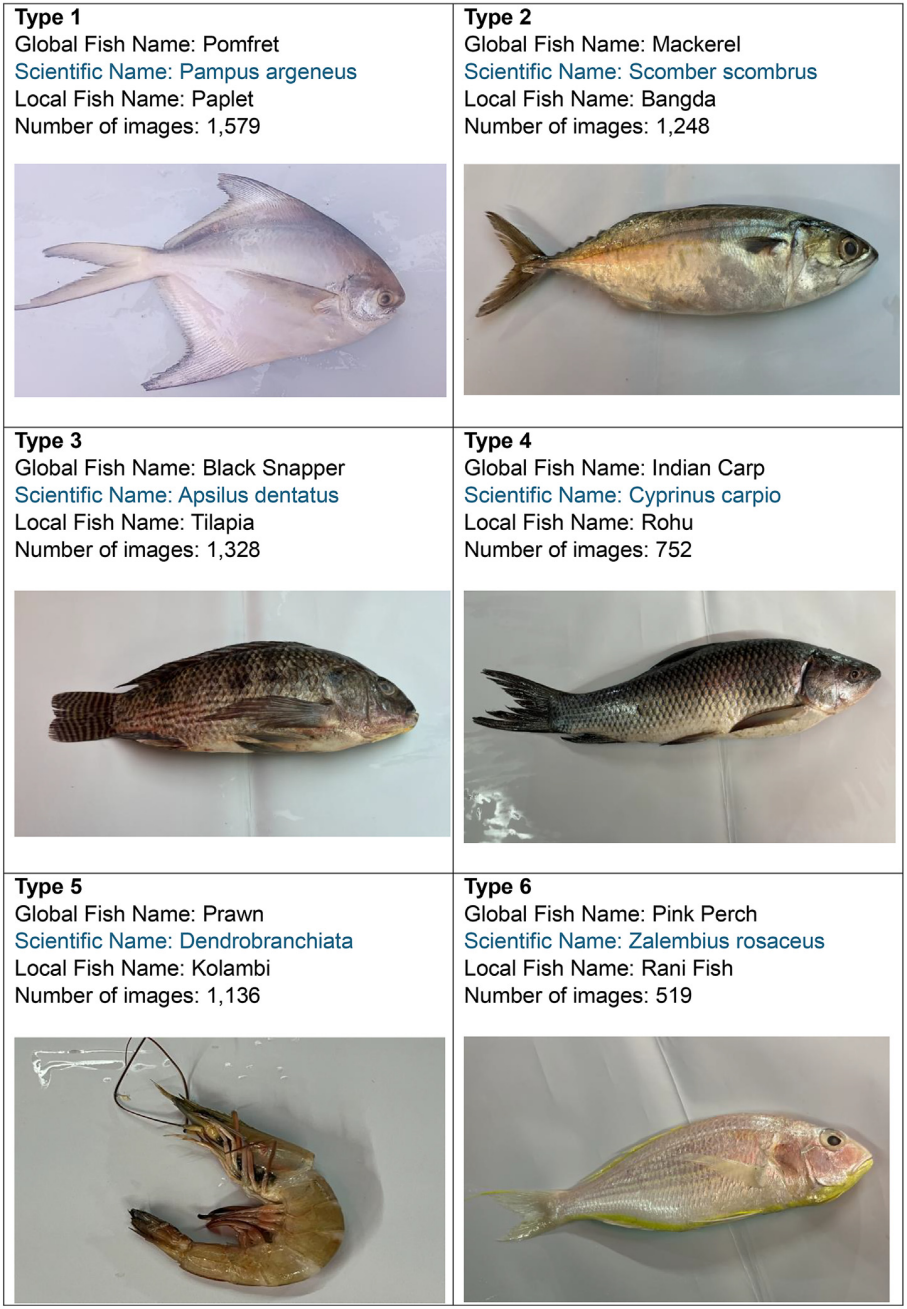

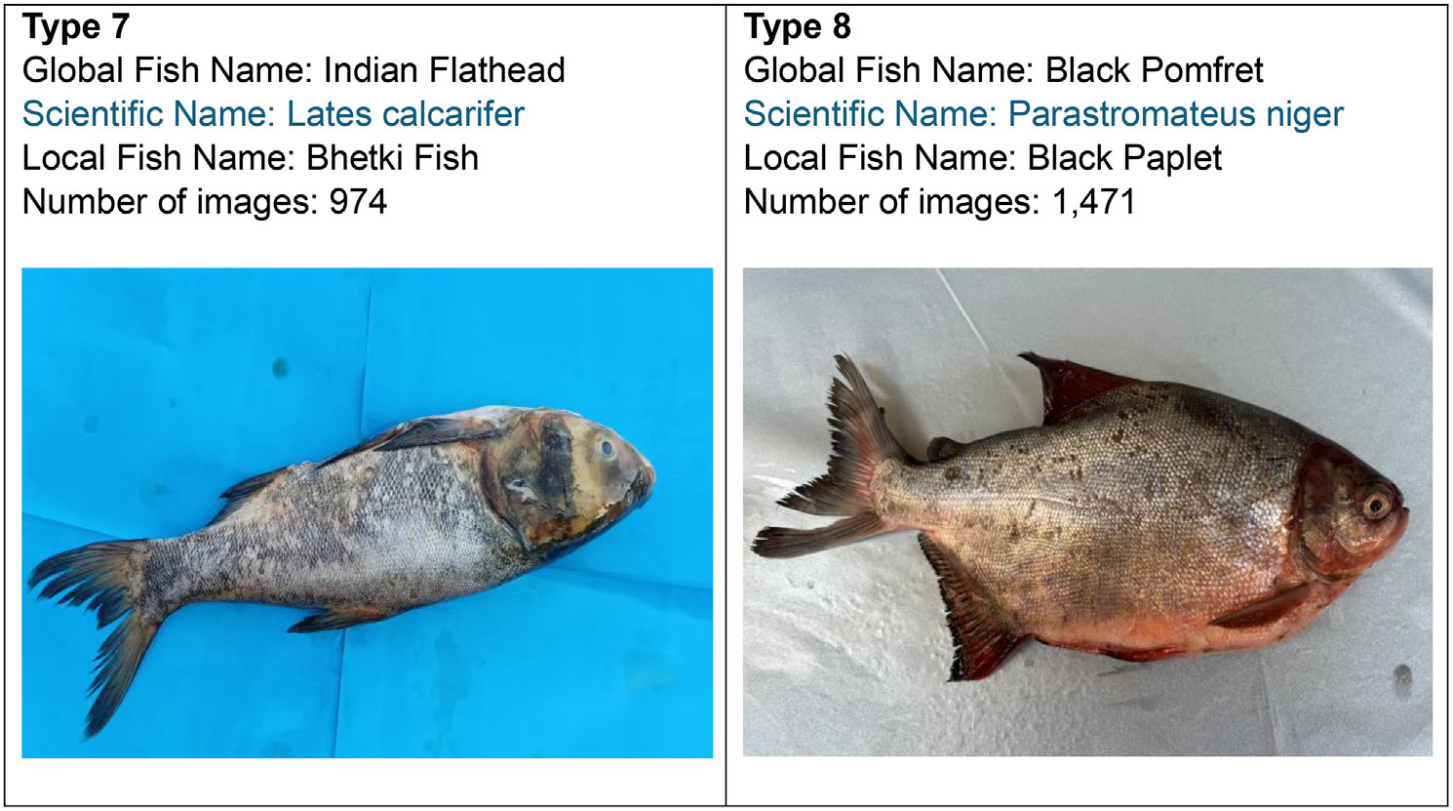
Sample images of each category.

The dataset captures the diversity of fish at a local market in Pune, India. It features over 8488 images across eight widely consumed types of fish in India: Pomfret (scientific name: Pampus argeneus, local name: Paplet), Mackerel (scientific name: Scomber scombrus, local name: Bangda), Black Snapper (scientific name: Apsilus dentatus, local name: Tilapia), Indian Carp (scientific name: Cyprinus carpio, local name: Rohu), Prawn (scientific name: Dendrobranchiata, local name: Kolambi), Pink Perch (scientific name: Zalembius rosaceus, local name: Rani Fish), Indian flathead (scientific name: Lates calcarifer, local name: Bhetki Fish) and Black Pomfret (scientific name: Parastromateus niger, local name: Black Paplet). Each species is stored in separate folders [2]. For a realistic representation, the images were captured with a consistent background but under various lighting conditions, both indoors and outdoors. Fig. 1 illustrates the folder structure of the dataset for easy navigation and Fig. 2 represents the sample images of each category.

## 4 Experimental Design, Materials and Methods

### 4.1 Experimental design

**Fig. 3:**
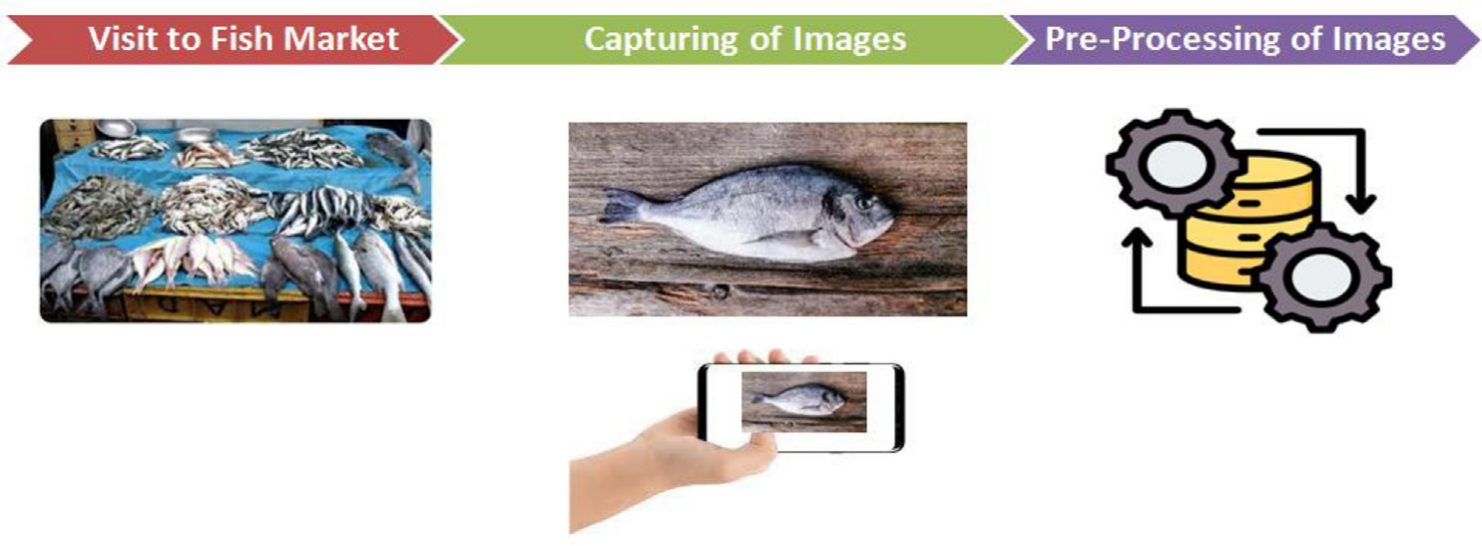
Steps in dataset creation.

Fig. 3 represents the image capturing process. We utilized high-resolution cameras on two smartphone models: the Realme 6i and the iPhone 6.

Initially, over 11,000 images were captured. Each image underwent a thorough examination to assess object clarity, and any images exhibiting blurriness were removed during the preparation phase to ensure high-quality results. Consequently, the final dataset comprises 8488 sharply focused photographs of various fish species. The processed images were meticulously organized into designated folders according to their respective classifications for convenient access. Fig. 3 illustrates the sequential steps involved in the dataset creation process, from the initial visit to the fish market to the subsequent processing of the captured images.

Photographs of fish were captured in a variety of settings from February to March. This process involved capturing pictures against the same backgrounds, in various perspectives, and with both artificial and natural lighting. Pre-processing included the use of Python script to resize all of the photographs to the same uniform standard size of 1024 × 768 pixels ensuring consistency across the dataset.

The initial step involved capturing images at the fish market. Subsequently, these photographs underwent a quality assessment and standardization procedure to ensure their inclusion in the final dataset.

### 4.2 Materials or specification of image acquisition system

The camera apparatus used to take the images, and the parameters of the final images are described in this section:

1. iPhone Mobile:
  a. Make and Model: iPhone 6 (Apple) Mobile.
  b. Rear Primary Camera: 48 MP, f/2.2
2. Realme 6i:
  a. Make and Model: Realme 6i Mobile.
  b. Rear Primary Camera: 48 MP, f/1.8

To ensure uniform image quality and compatibility throughout the dataset, all captured images were converted to a standard resolution of 1024 × 768 pixels and stored in JPEG format.

### 4.3 Method

**Fig. 4:**
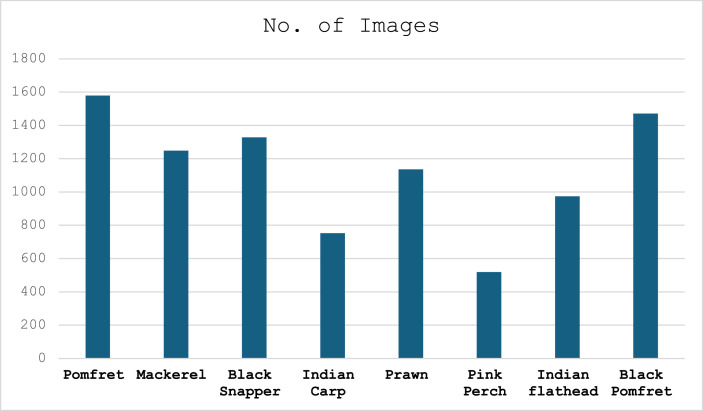
Distribution of fish species.

The Batch Image Resizer tool was applied for batch image resizing. Preprocessing techniques were used to make sure the quality of photographs for the dataset. Images are resized, stored, and then sequentially numbered. The final ready dataset is uploaded to Mendley Dataset Repository [1]. Fig. 4 shows the distribution of fish species in the dataset.

### 4.4 Highlighting the datasetʼs value

Table 1 represents a comprehensive comparison of existing Fresh Fish datasets for parameters such as number of images, number of species considered. Additionally, Table outlines limitations associated with each dataset. Some existing datasets include underwater images of fish. The presented dataset contains 8488 images representing 8 species of Indian seafood. In contrast, previously available datasets typically consist of only a few hundred images. The large number of images in this dataset makes it sufficient for training machine learning models for fish species detection and classification. While the primary focus is on species native to the Indian region, the dataset also includes a variety of widely consumed fish species.

**Table 1:** Comparative table for Fresh Fish dataset.

Sr. no.	Dataset ref. no.	Repository	Total no of images	No. of fish species considered	Limitation of dataset
1.	[3]	Mendeley Data	1208	1	The dataset consists exclusively of salmon fish.
2.	[4]	Mendeley Data	4392	8	Dataset of fish eye images only.
3.	[5]	Kaggle Data	Actual images: 359 Augmented images: 1000 per class	9	Dataset is 95 % augmented from original 5 % images.
4.	[6]	Roboflow Public Dataset	680	Not specified	Images of fish are taken randomly under sea water which is not widely consumed.
5.	[7]	Mendeley Data	Approx. 20 per species	6	Dataset is designed for classification based on fish visual features such as scale, head etc. Entire fish body images number is very low.
6.	[8]	Robotic vision lab dataset	Approx. 30–100 per species	8	Small dataset of sea fish species which are not widely known and consumed.
7.	[9]	Kaggle	Approx. 30–80 per species	31	Dataset size is less. Considered species are not widely known and consumed.
8.	[10]	Mendeley Data	Approx. 200–500 per species	8	Dataset does not include species those are widely consumed.
9.	[11]	Kaggle	1259 images	Not specified	The dataset of images taken under sea. Does not consider local fish species.
10.	[1] our dataset	Mendeley Data	8488	8	Indian widely consumed species are considered.

The dataset's potential is investigated to improve the performance of pre-trained, well-known machine learning models in the classification of fish using models like Xception, InceptionV3, EfficientNetB0, ResNet50 and VGG16.

The performance of the model is evaluated based on the accuracy achieved before and after training on the same dataset. The performance of both the pre-trained and post-trained models is assessed in the context of fish classification, as presented in Table 2.

**Table 2:** Comparison of ML models for pre-training and post-training on the fish dataset.

Machine learning model	Accuracy calculates before training on dataset	Accuracy calculates after training on dataset
Xception	45.32 %	91.41 %
InceptionV3	43.68 %	85.94 %
EfficientNetB0	49.23 %	92.53 %
ResNet50	51.11 %	93.21 %
VGG16	39.74 %	89.21 %

Results clearly indicates that accuracy improves significantly after training the same models over fish dataset. The dataset holds enormous potential for developing food identification software capable of recognizing fish dishes in images. Such applications could be valuable in automated food analysis in restaurants, dietary tracking programs, and recipe identification.

## Limitations

This fish dataset does not include all categories of fish and focuses primarily on widely consumed fish species in the Indian region.

## Ethics Statement

Our research aligns with Data in Brief's ethical considerations for datasets, as it does not involve live animal or human subjects. Thus, confirm adherence to ethical considerations.

## Credit Author Statement

**Prianka Paygude:** Conceptualization, Original draft preparation, **Namita Shinde:** Methodology, Software, **Amol Dhumane:** Methodology, Software**, Geeta Navale:** Data curation, Writing, **Prashant Chavan:** Writing- Reviewing and Editing, **Atul Kathole:** Visualization, Investigation, **Vijaykumar Bidve:** Supervision

## Data Availability

Mendeley DataIndian Seafood Fish Dataset (Original data)
